# Round the Hospitals

**Published:** 1919-11-15

**Authors:** 


### ROUND THE HOSPITALS.

The King's call to prayer on Armistice Day must reckon as one of the happiest inspirations of his reign. The nation cannot in the nature of things often be reminded that the sovereign is *ex officio* Head of the National Church, Defender of the Faith, but King George achieved by his simple mandate what ecclesiastical dignitaries have aimed at for centuries past in vain. He brought his people, high and low, rich and poor, learned and ignorant, irrespective of colour, race, class, or creed, to an act of spontaneous concentrated worship. They responded to his call, not merely with unanimity, but with joyful enthusiasm. In institutions for the sick the call sounded with thrilling intensity. In the utter stillness of the pause in ordinary occupations the names of fellow-workers, doctors and nursing sisters who had paid the last sacrifice in distant lands or on the sea crowded to memory, while the heart of many a sister went out to the man whom she had nursed back to life only to know that the life she saved had after all been given for the country. In most minds, after experience of the deep solemnity and unifying power of still worship, the hope was set

**ROUND THE HOSPITALS**—(*continued*).

stirring that this ceremony may yearly be observed among us in perpetuity.

The Day of Remembrance of our Glorious Dead was fittingly and impressively observed in the London Churches especially at the time of The Silence, and nowhere more so than at the Church of St. Martin-in-the-Fields in Trafalgar Square. Within the Church, services commencing at seven in the morning were held throughout Armistice Day to meet the need of the stranger and passer-by who cared to spend a few minutes in reflection and prayer. In the evening at eight o'clock the large congregation then gathered in the Church were methodically formed into procession, and, headed by the Cross, the Clergy, congregation, choir, and band, marched around Trafalgar Square to the singing of "Onward, Christian Soldiers," a procession in striking contrast to the green and red lights and whizzing fireworks displayed around. An assemblage of over 2,000 people returned to the Church for a service at nine o'clock, at which were given selections of music by the Band of the Welsh Guards. It is a Church which attracts many nurses, and with characteristic and kindly forethought the Vicar had reserved several rows of seats in the front and side aisle for nurses only, unable to leave their wards before nine o'clock. This anticipation of their comfort was greatly appreciated and fully availed of, the numbers attending more than filling the reserved seats. Amid that crowded Congregation the nurses present in their indoor uniforms and muslin caps constituted a stirring and powerful reminder of the great Ideals, and the Services rendered and still to be rendered by them to humanity, not principally for gain, but in fulfilment of Christ's teaching and Florence Nightingale's example.

In the Royal Warrant issued August 1917 which we re-printed in our issue of October 18, provision was made for granting additions to the pension of a disabled nurse "for any period during which she is prevented from earning her living by undergoing training in a technical institution or otherwise which, in the opinion of our Minister of Pensions, would benefit her." From this clause to the development of a satisfactory system of training for nurses unable by reason of their war service to continue in their profession was a far cry. But the preliminary arrangements are now so near completion that nurses pensioned on account of disablement, due to military service, who wish to be trained for some less arduous employment than nursing, are invited to send in their names to the Controller, Women's Training Branch, Ministry of Labour, St. Ermin's Hotel, Westminster, S.W. 1, all letters being marked "Disabled Nurse."

Candidates for training will have to obtain a certificate from the Ministry of Pensions' medical officer vouching for their fitness to undertake the new branch of work which they desire to take up, but this will be arranged for after application. The period of time allowed for training extends up to twelve months when necessary. The cost will be met by granting accepted candidates the difference between the pension actually in payment and the pension at the rate for the highest degree of disablement. In addition, the Ministry of Labour will undertake the payment of fees "within certain well-defined limits." We believe this policy to be just and economically sound. Women who have already made their mark in nursing, as these Army Sisters have done, are of exceptional value to the community. Technically, they have been trained to do but one thing and do it well, but potentially their powers are unlimited. Because, owing to their previous exertions, their physical powers no longer come up to the standard required in nursing, it by no means follows that they must be useless for the rest of their lives. Once the way is made clear to them to resume active work in some lighter employ, the latent forces which made them good nurses will re-adjust themselves, and we look to many of them to win distinction in the new career of their choice.

The Metropolitan Asylums Board has taken a ballot to ascertain whether the hospital nurses, in the new scheme for the shortening of their working hours, preferred a scheme based on the fifty-hour week with extended annual leave, or on the forty-eight-hour week. The result is that 8,066 balloted for the forty-eight-hour scheme and 578 for the fifty-hour scheme; and the view of the majority will prevail. Representations, however, have been made that the strict limitation of the hours to forty-eight in any one week would lead to frequent changes in the nurses attending on sick patients, and as the nurses have preferred their off-duty times aggregated at greater intervals than each week, it has been arranged to average the forty-eight hours a week over a period of one month.

*The Times* of the 7th instant contained a notice that Dr. Addison brought in on the evening of November 6 the Nurses' Registration (No. 2) Bill, which was read a first time. Six days have elapsed and no copies of this Bill have been obtainable as we go to press, though an expectant world is growing impatient at the delay which, we hope, will not be permitted to continue many days. Meanwhile Mrs. Fenwick took occasion, at a meeting held on Friday, November 7, where she described herself as the "Old War Horse and Leader," save the mark! to move a vote of thanks to Dr. Addison for the Bill of State Registration for Nurses, the reporter adding, "The impression given to the meeting, and it was understood to be so by all present, was that the Bill referred to was that of the Central Committee. The announcement was received with prolonged cheers and applause." Fancy any one who aspires to be regarded as "A Great Nursing Pioneer in History" talking through their bonnet and encouraging nurses, out of work for the moment, to join a Trade Union. Experience has taught working women, we are glad

**ROUND THE HOSPITALS—(*continued*).**

to find, that wise people do not count their chickens before they are hatched. The Central Committee have pursued the opposite course throughout. The run on last week's issue of *The Nursing Mirror* was a record.

Increasing interest and well-founded indignation are exercising the minds of many nurses in regard to threats and denunciations emanating from the same source as the attempt to establish a Professional Trades Union of Nurses. The oldest and most successful nurses' co-operation, of which the twenty-eighth report was issued in the spring of the present year, the Nurses' Co-operation, can show that the number of nurses now in this co-operation is 515, of whom 443 are on the general staff, 38 mental nurses, and 34 are extra members on probation. 6,055 cases were nursed in 1918, and the fees received by the nurses amounted to the sum of £57,286, the largest sum ever received. The nurses on the staff receive the whole of their earnings, subject only to a deduction of 5 per cent. in the case of those who joined before the end of 1908 and 7½ per cent. in the case of those admitted to the staff since that date. Founded in 1891, and incorporated in 1894 under the Limited Liability Acts, under Section 23 of the Companies Act, 1867, for many years the affairs of the Nurses' Co-operation were administered by a committee of voluntary members, who each subscribed one guinea a year. This committee consisted of ladies and gentlemen interested in nurses and members of the medical profession. As the demand for democratisation has become more and more a catchword with nurses the number of nurse representatives on the Committee of Management has exceeded the number of ordinary members of the Committee. No nurse or other person seeking to derive any profit or other benefit from the Society is eligible to be elected as a member.

Some £5,000, the accumulated savings through the thrift and careful management of the Committee in charge of the Nurses' Co-operation, includes all the savings in hand from the proceeds of the surplus derived, after paying all expenses, from the small commission deducted from the nurses' fees. This money is the property of the Association, and cannot be paid out or distributed amongst the members of the Society, but shall be given or transferred to some other institution or institutions having objects similar to the objects of the Society, to be determined by the members of the Society at or before the time of its dissolution. It is incredible that the nurse-members whose affairs have been so excellently administered under its constitution for some twenty-six years, with such splendid financial results, should consent to have their organisation and connection suddenly terminated by winding-up this Society. We mention the matter that every nurse-member may have her attention directed to it, and that she may become alert to the defence of her own Association and interests, which are threatened by a type of parties, who have already shown the nursing body more than once or twice during a long period of years, that, it is the nurses' money they want, almost always, at the bottom, whatever else they may start out to accomplish.

A strong demand for paying wards in hospitals or nursing homes under public management is making itself felt in Manchester, where the claims of "middle-class martyrs" are being urged under the somewhat dubious argument that "at present illness is a luxury, only to be indulged in either by the very rich or the extremely poor." Nurses are very much interested in this question, for there can be no doubt that the extension of facilities for institutional treatment to patients able to afford moderate fees, would curtail the private nurse's *clientèle*. It would, however, at the same time open up a new field of employment, and with an eight-hour day, a just and liberal scale of salaries and suitable accommodation or allowances, the nurses at municipal nursing homes and private hospitals ought to be very much better off than private nurses putting in a twelve-hour day or night among the middle classes, whose poverty, though veiled, often reacts acutely on their nurses.

One of the most pressing difficulties which present themselves in the organisation of district nursing in great cities arises out of the suddenness with which emergencies occur. The normal work of the nurses becomes doubled or trebled at a moment's notice, with the result that, as in last year's outbreak of influenza, the system breaks down and sweeps many of the nurses along with it. The Central Council for District Nursing in London, which has already, since its formation in 1914, accomplished much, has set itself to safeguard the public and its own nurses by the establishment of a register of emergency nurses, from which the various nursing associations can draw in times of exceptional sickness. Particulars of the qualifications and duties of the emergency nurses will be found in our advertisement columns. The position of emergency nurse is well suited to demobilised sisters awaiting fresh work; to others who have retired from active work but are willing to serve for part of the year, as required; for some who have married but are able to respond to the call of duty for short periods when urged by special need for their services. We confidently expect that there will be a hearty response to the call of the Council, and that the Register of Emergency Nurses will take its place among the indispensable branches of the national nursing service.

Some two years before war broke out the Red Cross Societies of the world, meeting at Washington in an international conference, decided to establish a medal, to be called the Florence Nightingale Medal, to be distributed to six trained nurses annually or in alternate years, selected by recommendation from various Red Cross Societies as most worthy of the honour. Before the Committee in

**ROUND THE HOSPITALS**—(*continued*).

Geneva, appointed to carry out this decision, had completed their initial labours, the war rendered a long postponement necessary. However, the committee have now resumed their operations and have informed the British Red Cross Society that 50 of the Florence Nightingale medals are to be distributed in January 1920. The British Red Cross invite doctors, matrons, and nurses to assist in the award of these medals by sending in the names of nursing sisters who have proved themselves, especially during the war, to be exceptionally worthy of recognition. In sending in the names care should be taken to give very legibly the name and address, with details of the professional qualifications of the lady selected for mention. The services which entitle her to distinction must be clearly recounted, with date and locality at which they were rendered. Lastly, the names of the sister's superior officers, or other officials who can personally vouch for the accuracy of the information given must be supplied. Recommendations should be sent marked on the outside "Nightingale Medal," to the Secretary, British Red Cross Society, 83 Pall Mall, S.W. 1, and must be received before the last day of November.

---

THE proceedings before the sub-committee appointed to consider the advisability of conferring military rank on American Army nurses, as reported in the *American Journal of Nursing* for October, contains some evidence of great interest to all British nurses. Miss Parsons, Nursing Superintendent of the Massachusetts General Hospital, and chief nurse at the United States base hospital at Bordeaux is strongly contending for military rank. The lack of it, she declares, decreased the efficiency of her colleagues, and was very humiliating in a foreign country. In particular, she stated that the British nurses have a very dignified position—"It is so dignified that it is generally supposed they have rank." The chairman objecting that British nurses do not have rank, Miss Parsons continued: "They do not have rank *per se*. But their status has been so clearly defined and so carefully upheld through the influence of Queen Alexandra, for whom they have been named, that it has been equivalent to rank in the dignity, respect, and obedience it has commanded. Because their position is established on so high a plane, ninety-nine men out of a hundred will tell you that the English nurses have rank." It is sometimes very encouraging, as well as wholesome, to see ourselves as others see us. And it is well British nurses should be reminded by nurses on the other side of the Atlantic of what they owe to the continuous, untiring labours of Queen Alexandra. All the same, Miss Parsons is quite wrong in her view that soldiers imagine British Army nurses to have rank: soldiers are not such fools where matters of rank are concerned.

---

NURSES in town should not miss the *Englishwoman* Exhibition of Arts and Crafts, open from November 12 to 22, at the Central Hall, Westminster. Not only is it an opportunity for securing novelties of all kinds for Christmas gifts, but it is an object-lesson on the uses of a paying hobby to professional women.

---

AN inquest was held at Bethnal Green, November 10, on the body of a dock labourer named Charles Mace, who met his death by jumping from a second-floor window at the Victoria Park Chest Hospital, 35 to 40 feet from the ground. Evidence was given that Mace was the sole occupant in a small ward, having developed mania and been isolated for that reason. A probationer named Bertha Ward was in charge of the patient and saw him get out of bed about 11 o'clock. After a close struggle with her he got away, ran to the window and opening the lower sash partly dropped out. Nurse Ward very pluckily held on to him by his arms and clothing and shouted to the gardener and porter for help. Unhappily no one heard her calls and eventually the weight proved too great for her strength and the patient slipped through her hands and fell to the ground. The verdict was "Suicide while of unsound mind." The authorities of the hospital must, we feel sure, be dissatisfied with arrangements which placed a young probationer in charge of a delirious patient with no facilities for summoning assistance. Bertha Ward is a probationer of promise who will be heard of again if she lives and continues nursing. She is evidently a brave, quick-witted woman, with courage and resource. The Committee should suitably mark their sense of their appreciation and thanks to Probationer Ward.

---

A REMARKABLE indictment of the system pursued at the Foundling Hospital has recently been published by Messrs. Heedler, under the title of "The Child She Bare," by a Foundling. The picture of life in an institution, however well managed, as seen from within, is valuable, for few, even of those who devote all their energies to the care of orphaned children, are able to realise things from the point of view of their *protégés*. But the point we wish to bring before our readers is the official attitude towards illegitimacy. It seems almost inconceivable that a mistress should be found in such a home who could "frequently" call the little girls "children of shame," but we can more readily understand that the children may be allowed to leave the institution in adolescence without any light being thrown on their peculiar position, with the result that in their first situations they may be cruelly disillusioned and made subjects of coarse abuse. How best to tell illegitimate children the truth about themselves is a matter for very serious consideration, but we have no doubt at all that it ought to be undertaken. Sir Arthur Pearson's attitude towards blindness is undoubtedly the bravest and wisest to adopt. Their status is a handicap but not an irremediable calamity, and the knowledge that life is thereby made more difficult for them should be used as a stimulus to aid in overcoming circumstances.

